# Sodium valproate in pregnancy: what are the risks and should we use a shared decision-making approach?

**DOI:** 10.1186/s12884-018-1842-x

**Published:** 2018-06-01

**Authors:** Alastair Macfarlane, Trisha Greenhalgh

**Affiliations:** 1 Academic Foundation Year 1 Doctor, Barnet Hospital, Wellhouse Lane, Barnet, EN5 3DJ UK; 2 Department of Primary Care Health Sciences, University of Oxford, Radcliffe Primary Care Building, Woodstock Road, Oxford, OX2 6GG UK

**Keywords:** Sodium valproate, Pregnancy, Teratogenicity, Epilepsy, Bipolar disorder, Shared decision-making

## Abstract

**Background:**

Despite significant teratogenic risks, sodium valproate is still widely prescribed in many countries to women of childbearing age, as a mood stabiliser in bipolar disorder and also in epilepsy. The UK has recently banned valproate use in women who are not in a pregnancy prevention programme. Whilst this ruling reflects prevailing clinical practice, it also highlights an ongoing debate about when (if ever) a woman who is or could become pregnant should be allowed to choose to take valproate.

**Main body:**

We review the benefits and harms of drugs available for bipolar disorder and epilepsy in women of childbearing age, with a particular focus on teratogenic risk. We speculate on hypothetical rare situations in which potential benefits of valproate may outweigh potential harms in such women. We also review the literature on shared decision-making – on which there is now a NICE guideline and numerous evidence-based decision tools. Drawing on previous work by experts in shared decision-making, we offer a list of ‘frequently asked questions’ and a matrix of options to support conversations with women about continuing or discontinuing the drug in (or in anticipation of) pregnancy. We also consider whether shared decision-making is an appropriate paradigm when considering whether to continue a teratogenic drug.

**Conclusion:**

We conclude that because valproate in pregnancy remains the subject of such debate, there is scope for further research – not only into the relative efficacy and safety of alternatives to it – but also into the dynamics of communication and shared decision-making in this situation.

## Background

In July 2017, almost 60 years after the thalidomide tragedy [1], the French National Agency for the Safety of Medicines and Health Products (ANSM) imposed a nationwide ban on the use of sodium valproate in pregnancy, on the grounds of teratogenicity [2]. Guidelines produced by the UK Royal College of Obstetricians and Gynaecologists in 2016 recommended avoiding this drug in any woman of child-bearing potential [3] (reflecting a previous NICE guideline published in 2014 [4]). More recently, regulatory bodies have tightened their stance significantly. In February 2018, for example, the Pharmacovigilance Risk Assessment Committee of the European Medicines Agency recommended that valproate should not be used in pregnancy unless the woman has a form of epilepsy that is unresponsive to other anti-epileptic drugs, and also that the drug should not be prescribed for women of childbearing age who are not enrolled in a pregnancy prevention programme [5]. In April 2018, the UK Medicines and Healthcare Devices Regulatory Agency endorsed this recommendation [6].

Sodium valproate is a medication licensed for both epilepsy [7] and bipolar disorder [8], and is also used off label for a range of indications including migraine prophylaxis [5]. Whilst its use in epilepsy is falling in the UK as the use of third-generation anticonvulsants increases [9, 10], its use for bipolar disorder has been increasing, especially among women of childbearing age [11]. In Ireland, recent data corroborate these findings [12], which necessitate increased surveillance and an improved understanding of alternatives [13].

Bipolar disorder is a psychiatric condition characterised by alternating periods of elated mood and depression [14]. The condition is a significant public health problem worldwide, and remains a challenge for patients and clinicians [15]. Systematic reviews examining the epidemiology of bipolar disorder show that it is associated with markedly increased suicide and self-harm rates [16], substance abuse [17] and other psychiatric morbidities [18–21]. Given the female preponderance and young age of onset of bipolar disorder (17.5 years) [22], women of childbearing age make up a significant proportion of patients. Management of bipolar disorder is difficult in this group, as many mood stabilisers have either been shown to be teratogenic or have unknown effects in pregnancy (see below) [23].

Epilepsy is a condition characterised by abnormal excessive synchronous neuronal activity in the brain causing seizures [24], and is associated with a variety of neurobiological, psychological, social and cognitive consequences. The prevalence of active epilepsy in adults is 5–10 per 1000, and is influenced by many factors, both genetic and environmental [25]. One risk factor for seizure activity is oestrogen [26], hence pregnancy can increase the seizure rate [27]. Information from EURAP, the International Registry of Anti-Epileptic Drugs and Pregnancy, suggests that 20% of pregnant patients with epilepsy were treated with valproate from 1999 to 2004, despite knowledge of its teratogenic risks [27]. A more recent study in the UK showed that valproate made up 25% of anti-epileptic drug prescriptions in pregnancy [28].

This article will explore the benefits and harms of sodium valproate and its alternatives in current or planned pregnancy in the context of a tightening regulatory system for this drug. We will suggest questions that should guide decision-making for these patients, and address the crucial issue of whether and how patients can be involved as democratic partners in such decisions.

## Main text

### Mood stabilisers in bipolar disorder

Mood stabilisers are the mainstay of pharmacological management in patients with bipolar disorder. Although there is some contention about what exactly constitutes a mood stabiliser [29], the defining feature is that these drugs improve both manic and depressive symptoms without significantly worsening either polarity [8]. Some drugs may be of benefit in patients with bipolar disorder but are not classed as mood stabilisers because of their ability to precipitate mania (e.g. some antidepressants) or worsen depression (e.g. some antipsychotics).

Table 1 shows the main classes of mood stabiliser used in the treatment of bipolar disorder. Sodium valproate, a mood stabiliser of the anticonvulsant class, is commonly prescribed for the treatment of mania and the prophylaxis of bipolar disorder [30]. A systematic review assessing valproate efficacy in acute mania showed that it has a has a number needed to treat (NNT) of between 2.3 and 4.3 [31], and may be used in patients who have either failed to respond to lithium [32], or those who do not tolerate it [33]. Prophylactically, the NNT to *prevent* manic and depressive episodes respectively is 21.3 and 10.5 [34].

**Table 1:** Main classes of mood stabiliser (and alternatives to valproate in the treatment of bipolar disorder), mechanisms of action and side effects (not including foetal or maternal risks). Compiled from various sources [10, 33, 34, 91–100]

Class	Medication name	Proposed mechanism(s) of action	Side effects
Mineral	Lithium	Enhances serotonergic neuron activity, inhibits pAp-phosphatase enzyme, interacts with nitric oxide signalling activity	Common: GI upset, fine tremor, polyuria, polydipsia, metallic taste in mouth, ankle oedema, weight gain. Chronic: renal toxicity, hypothyroidism.
Anti-epileptics	Sodium valproate	GABA potentiation, blocks voltage gated sodium channels, epigenetically inhibits histone deacetylase	Common: GI upset, hyperammonaemia (causing nausea), weight gain, tremor, hair loss with curly regrowth. In women: polycystic ovarian syndrome, hyperandrogenism. Rare: fulminant liver failure.
	Lamotrigine	GABA potentiation, suppresses glutamate release, inhibits serotonin reuptake	Common: tremors, dizziness, tiredness, loss of co-ordination, menstrual disturbance, dry mouth, sleep problems.
	Carbamazepine	Blocks voltage gated sodium channels	Common: dizziness, diplopia, drowsiness, ataxia, nausea, headaches, dry mouth, oedema, hyponatraemia, erythematous rash, sexual dysfunction. Rare: agranulocytosis.
Atypical antipsychotics	Risperidone	Dopaminergic (D_1–5_) receptor antagonist, serotonergic (5-HT_2A/C_) receptor antagonist	Common: sexual dysfunction (hyperprolactinaemia). Long term: movement disorders (e.g. tardive dyskinesia, akathisia, parkinsonism), increased risk of cardiovascular disease. Rare: neuroleptic malignant syndrome
	Olanzapine		
	Quetiapine		
	Aripiprazole	Dopaminergic (D_2_) and serotonergic (5-HT_1A_) receptor partial agonist	Common: weight gain, headache, agitation, insomnia, gastrointestinal effects, disinhibition.

Valproate has a number of side effects other than risks to the mother and foetus, which are summarised in Table 1. In any patient, these side effects must be weighed against the significant risks associated with untreated mania or bipolar disorder, including suicide [16].

A high proportion of patients with bipolar disorder will face the scenario of needing to manage their illness during an anticipated or current pregnancy [22]. This raises a very difficult clinical issue: managing the mental health needs of the mother whilst minimising the teratogenic risk to the developing foetus. Bipolar disorder per se does not increase the risk of malformation or foetal death [35], but several mood stabilisers are associated with major teratogenicity.

All psychotropic medications cross the placenta [36]. As well as the recognised risk of teratogenicity – which occurs in the first trimester during organogenesis – there are further ways in which these medications may adversely affect a pregnancy. Viguera et al. categorise the effects of psychotropic medication use in pregnancy into: [1] obstetric complications (such as low birth weight), [2] perinatal complications (occurring shortly after birth) and [3] long-term neurological and behavioural sequelae (such as autism) [23]. Table 2 summarises the main foetal and maternal risks associated with mood stabilisers.

**Table 2:** Foetal and maternal risks associated with selected mood stabilisers. Compiled from various sources [50, 53, 78, 79, 101–106]

Medication	Risks to offspring associated with use in pregnancy	Maternal risks associated with use in pregnancy
Lithium	Severe toxicity in newborn. There are limited and conflicting data regarding the risk of cardiovascular malformations (including Ebstein’s anomaly) following lithium exposure in utero. A large cohort study in 2017 showed that the relative risk was still elevated (1.7), and also dose dependent, but lower than previously thought. Absolute risk remains low (< 1/1500). Non-teratogenic associations include low birth weight, cyanosis, bradycardia, GI bleeding, polyhydramnios, seizures.	Renal lithium clearance rises during pregnancy, so levels need to be monitored regularly to maintain therapeutic levels.
Valproate	Significantly elevates the risk of major defects (7 times higher). These include spina bifida, atrial septal defect, cleft palate, hypospadias, polydactyly and craniosynostosis. See Table 4 and main text for further details. Non-teratogenic associations include case reports of intra-uterine growth restriction, infant hepatic toxicity and foetal distress during labour. Neurodevelopmental associations – foetal exposure to valproate in utero is associated with 1.7 times risk of autism spectrum disorder.	Increased hepatic clearance of valproate and increased apparent volume of distribution cause lower maternal levels of the drug.
Carbamazepine	Risk of major congenital abnormalities increased 1.8 times, including malformations of neural tube, urinary tract and cardiovascular system, and cleft palate.	Crosses placenta and lowers maternal serum levels, so doses may need to be increased.
Lamotrigine	Conflicting evidence on the risk of malformations, especially regarding dose response. Evidence emerging that it appears to be a relatively safe drug in pregnancy.	Crosses placenta and lowers maternal serum levels, so dose may need to be increased. Dizziness, diplopia and ataxia have been reported following these dose increases in pregnant women.
Atypical antipsychotics	Most do not appear to significantly increase malformation rate. Risperidone requires additional study.	Crosses placenta and lowers maternal serum levels, so doses may need to be increased.

### Anti-epileptic drugs

In epilepsy as in bipolar disorder, valproate is not the only available drug for women of childbearing age. And again, there is a somewhat complex picture of efficacy and safety in the different alternatives. Schmidt and Schachter recently reviewed drug treatment for epilepsy [10], and highlighted that epilepsy is not a single condition but an umbrella term encompassing different underlying pathologies and clinical manifestations. Different kinds of epilepsy respond to different drugs and in the 20–30% of cases that are refractory, multiple drugs need to be used in combination. Getting the right drug for a person with epilepsy involves balancing benefits and side effects as well as taking account of lifestyle issues and personal preferences [37].

Table 3 summarises the main anti-epileptic drugs that could be offered to a woman of childbearing age. Whilst valproate (first licensed in 1967) is no longer the commonest anti-epileptic drug prescribed, it remains one of the most effective. It is still used in emergency settings to control focal and generalised seizures; it may be effective when newer anticonvulsants have not worked; and it is the recommended first-line therapy for complex partial seizures.

**Table 3:** Selected anti-epileptic drugs (and alternatives to valproate in the treatment of epilepsy), adapted from Schmidt and Schachter [10]

Class of drug	Name of drug	Proposed mechanism	Side effects	Additional information
1st generation	Phenytoin	Sodium channel blocker	Enzyme inducer (hence interaction with other medications), skin hypersensitivity	First line for focal and generalised seizures with focal onset
	Ethosuxamide	T-type calcium channel blocker	Gastrointestinal side effects, insomnia, psychosis	First line for absence seizures
2nd generation	Carbamazepine	Sodium channel blocker	Enzyme inducer, skin hypersensitivity	First line for focal and generalised seizures with focal onset
	Valproate	GABA potentiation, blocks voltage gated sodium channels, epigenetically inhibits histone deacetylase	GI upset, weight gain, tremor, hair loss with curly regrowth, teratogenicity (see Table 4) In women: polycystic ovarian syndrome, hyperandrogenism Rare: fulminant liver failure	First line for focal and generalised seizures, no skin hypersensitivity, no newer drugs have been shown to have higher efficacy
3rd generation	Vigabatrin	GABA potentiation	Visual defects, weight gain, seizure aggravation, encephalopathy	Use in infantile spasms, adjunct in complex partial seizures
	Lamotrigine	GABA potentiation, suppresses glutamate release, inhibits serotonin reuptake	Tremor, dizziness, tiredness, loss of co-ordination, menstrual disturbance, dry mouth, sleep problems	First line for focal and generalised seizures, lower efficacy than valproate for absence seizures
	Oxcarbazepine	Sodium channel blocker	Enzyme inducer, hyponatraemia, skin hypersensitivity	First line for focal and generalised seizures with focal onset
	Gabapentin	Calcium channel blocker	Weight gain, psychosis, seizure aggravation, tiredness, dizziness	Adjunctive use only, used in focal and generalised seizures with focal onset
	Levetiracetam	SV2A modulation	Tiredness, dizziness, behavioural problems	First line in focal and generalised seizures with focal onset and myoclonic seizures.
	Topiramate	GABA potentiation, glutamate inhibition, sodium/calcium channel blocker	Tiredness, dizziness, skin hypersensitivity, weight loss, teratogenicity	First line for focal and generalised seizures

Schmidt and Schachter point out that whilst two in every three women with epilepsy who become pregnant remain seizure free throughout their pregnancy, antiepileptic drug dosages may need to be adjusted as the pregnancy progresses, particularly when seizures occur in the first trimester. Women taking lamotrigine, levetiracetam, topiramate, and oxcarbazepine may need an increase in dose to compensate for pregnancy-related increase in clearance of these drugs so as to reduce the risk of breakthrough seizures. These authors also highlight the small increased risk that the children of women who take an antiepileptic drug during pregnancy will be small for gestational age and have a lower Apgar score.

### A woman of childbearing age taking valproate…

There are two conceivable scenarios when a woman taking valproate would have the dilemma of continuing it during pregnancy: [1] she is *planning* to become pregnant whilst taking valproate; or [2] she has *already* become pregnant whilst taking valproate. Both these scenarios are currently fairly common in both primary and secondary care [38]. In addition to the fact that both bipolar disorder and epilepsy are common in women of childbearing age [15], patients suffering from bipolar disorder are at increased risk of unintended pregnancy for various reasons, including sexual disinhibition associated with mania [30].

Within these two scenarios, several factors might affect the clinical outcome for both mother and child: the severity of the condition (including frequency and duration of relapses), co-existing medication (including the need for combination therapy to control the bipolar disorder or epilepsy), drug and alcohol use, co-morbidity, sociocultural factors, level of social support; and also (in the second scenario) the stage at which the pregnancy was confirmed.

Deciding whether or not to continue valproate in these situations requires a complex risk assessment. Many studies over the years have assessed the sequelae of continuation and discontinuation of valproate – both to mother and child. We review these below.

### Risks of continuation

Prospective controlled trials on the effects of valproate during pregnancy are limited for obvious reasons, but cohort studies have shown that women who reported using the medication during the first trimester of pregnancy had a seven-fold higher risk of congenital malformations compared to the baseline rate of 1.62% [39]. Indeed, the relative risk of valproate on major defects is so high, and these defects are so characteristic, that the term ‘foetal valproate syndrome’ has been described [40]. Reasons for the teratogenic effects are not fully understood, but possibly involve epigenetic effects, including the inhibition of histone deactylase with associated changes in gene expression [41], increases in foetal oxidative stress, or the antagonism of folate required for DNA synthesis [42].

As well as its teratogenic effects, valproate may lead to problems after birth, including immediate withdrawal effects such as jitteriness [40]. Valproate therapy during pregnancy has been shown to correlate with longer-term neurodevelopmental problems leading to repetitive behaviours, impaired communication and social isolation [43, 44], as well as reduced IQ [45]. These autistic-like traits have been demonstrated in both animal models [46, 47] and human studies, affecting around 40% of children exposed to valproate [48, 49]. Actual diagnoses of autism spectrum disorder are lower, however, at around 4% [50]. Although the mechanisms underlying these are yet to be elucidated, there is a possible link with lower cell density in the cerebellum [51], associated with mutations in the PTEN gene [52].

The risk of valproate use in pregnancy on specific malformations has been quantified. Table 4 shows the odds ratio of different malformations based on an extensive review of the research literature [53]. Risk of malformation increases with drug dose and with combination therapy [54–56].

**Table 4:** Odds ratios and absolute risk of congenital malformation with sodium valproate (adapted from Jentink) [53]

Condition	Odds ratio (median and range) in offspring of mothers who took valproate in pregnancy	Absolute risk
Spina bifida	12.7 (7.7–20.7)	0.6%
Atrial septal defect	2.5 (1.4–4.4)	0.5%
Cleft palate	5.2 (2.8–9.9)	0.3%
Hypospadias	4.8 (2.9–8.1)	0.7%
Polydactyly	2.2 (1.0–4.5)	0.2%
Craniosynostosis	6.8 (1.8–18.8)	0.1%

Considerations about continuation should also address drug-related risks to the mother. Full blood count and liver function tests, for example, should be measured regularly to rule out blood dyscrasias or liver pathology [30].

### Risks of discontinuation

There are risks associated with discontinuing valproate in a patient whose bipolar disorder or epilepsy is well controlled. Viguera et al. found that pregnant women with bipolar disorder who were euthymic at conception but stopped mood stabilisers were twice as likely to relapse than those who continued mood stabilisers, and the median time until first recurrence was four times shorter [57]. If the mood stabiliser was discontinued abruptly, recurrence latency was eleven times shorter. Most studies of mood stabiliser discontinuation during pregnancy have been on lithium withdrawal [58–60]; specific evidence on discontinuing valproate in pregnancy is sparse. However, evidence for valproate withdrawal outside of pregnancy illustrates similar trends: relapse rates are high, especially during abrupt withdrawal [61–63], and there is even a case report of one patient being treated for epilepsy developing new-onset mania following valproate withdrawal [64].

Discontinuation can also be problematic in epilepsy. Observations from the EURAP study showed that withdrawal of valproate in the first trimester (when it is most teratogenic) was associated with a significantly higher rate of generalised tonic clonic seizures (33%) compared to when it was continued (16%) [65]. More striking, however, is that the rate of seizures was also elevated (29%) when valproate was switched to another anti-epileptic medication.

Another consideration for bipolar disorder patients should include the risks of puerperal psychosis. Prevalence of this condition in the general population is about 0.1–0.25%, but may be up to 50% in women with bipolar disorder [66]. Hospitalisation for psychiatric morbidity predicts the risk of puerperal psychosis [67], so untreated bipolar disorder may not just affect the mother *during* pregnancy, but also in the weeks afterwards.

Psychosis during the perinatal period must be viewed in a broader context: leaving it untreated can cause harm to the mother through poor self-care, increased drug and alcohol use, non-attendance for obstetric care and impulsive acts [30]. In severe cases, there may be direct harm to the child through untreated maternal psychomorbidity, including neglect or even infanticide [68, 69]. A 20-year study on puerperal psychosis in Austria found that out of 96 patients, six died from suicide, with three ‘extended suicide attempts’ leading to two cases of infanticide [70]. Furthermore, severe mental health issues during and soon after pregnancy can adversely impact the child’s emotional, cognitive and physical health later in life [71]. The child may be removed from the care of the mother for safeguarding reasons, leading to problems with bonding.

In sum, whilst there are good reasons to minimise the use of sodium valproate in pregnancy, it is theoretically possible that in some individual cases, the risks of discontinuing the drug could outweigh the benefits.

### Pregnancy prevention programmes and valproate

A ‘pregnancy prevention programme’ is defined by the new UK regulations on valproate prescribing as follows [5]:

1. There must be an assessment of the woman’s potential to become pregnant and pregnancy tests before and during treatment.
2. The woman must be offered counselling about the risk of valproate to her unborn child and the importance of using effective contraception while taking the drug.
3. Review by a specialist is now mandatory, and women taking valproate will be required to have annual specialist reviews including completing a risk acknowledgement form.
4. The packaging for valproate will carry a visual warning of the risks associated with pregnancy. Pharmacists will be required to discuss the risks and issue a warning card every time they dispense valproate to women of childbearing age.

Many of these steps reflect what has become accepted good practice in several countries over the past few years [4, 72], but they are now becoming mandatory in the UK. Clinicians generally avoid starting women of childbearing age on valproate, but when this is viewed as clinically unavoidable, information and a discussion about risks, along with an offer of contraception, are a core component of care.

Whilst the new requirements are therefore in line with current *recommended* best practice, there is also evidence suggesting an evidence-practice gap. For example, a recent study in the UK showed that around half the women taking sodium valproate were unaware of its potential to damage the foetus [73]. A literature review on management of women with substance-use disorders found that unplanned pregnancy was common but also that access to long-acting reversible contraception through integrated contraception services was an effective approach to targeting this problem [74]. We could learn from approaches taken in low-income countries, such as the implementation of post-abortion contraception in Zimbabwe, which has significantly decreased unplanned pregnancy rates in women with mental health conditions [75].

The above measures will only be relevant, of course, if the woman finds it acceptable to prevent pregnancy. In many women, there comes a time when pregnancy is desired. The clinician should explain to the women that whilst it is never possible to promise a healthy baby, the chances of this happening will be best if the pregnancy is carefully planned, including adjusting medication in the pre-conception period – which in the UK must (with rare exceptions) involve discontinuation of valproate. Women should be encouraged to report a pregnancy as soon as the test is positive – and reassured that the doctor will not be judgemental even if the pregnancy was “unplanned”.

Patients with bipolar disorder who are already taking valproate and are planning pregnancy have two options: (1) withdraw valproate slowly prior to conception, with close monitoring of their mental health status, or (2) switch to a lower-risk mood stabiliser. The first option needs to be carried out cautiously to minimise risk of relapse, and the decision to do this should take into account past history of relapse, co-existing medications and any protective or predisposing risk factors.

The second option reduces the risk of maternal psychomorbidity during pregnancy, but with a higher risk to the foetus. Although the most commonly prescribed mood stabilisers (lithium, carbamazepine and valproate) are associated with foetal abnormalities – albeit to differing extents – current evidence on lamotrigine and atypical antipsychotics is more favourable [36].

Various studies have looked at the safety of lamotrigine use in pregnancy, particularly regarding its dose. Tomson et al.*..*, using the EURAP data, found differences in the malformation rates in patients given < 300 mg/day and ≥300mg/day (2 and 4.5% respectively) [76]. Campbell et al, using data from the UK Epilepsy and Pregnancy Register, found no significant relationship between dose and malformation rate, and concluded that whilst ‘lamotrigine has a favourable profile compared with valproate for adverse pregnancy outcomes, the requirements for seizure control should not be overlooked’.

Atypical antipsychotic drugs may be used in the management of bipolar disorder and are typically recommended for short-term use [77]. In a review of treatment of bipolar disorder in pregnancy, Grover and Avasthi cite numerous studies with differing findings on the safety profiles of olanzapine, risperidone, quetiapine, amisupiride, ziprasidone, aripiprazole and clozapine, but conclude that these drugs are nonetheless safer than lithium or valproate in pregnancy [36]. A recent large cohort study in the United States showed that antipsychotic use early in pregnancy does not ‘meaningfully increase the risk for congenital malformations overall or cardiac malformations in general’ [78]. Risperidone carries a small increased risk, and requires additional study.

A recent letter in the BMJ highlighted that as the warnings regarding valproate use in pregnancy are justifiably being strengthened, it is very important to search for safer alternatives [79]. Nevertheless, we should not underestimate the difficulty of interpreting observational data on medication effects during pregnancy.

### How to minimise valproate use during pregnancy

The most difficult clinical decisions may arise when a patient using valproate presents when already pregnant. Presentations later in pregnancy and those in women on higher doses of valproate are associated with an increased risk of foetal abnormalities [56]. Current recommendations are to withdraw the drug if possible, and especially during the first trimester [49]. However, in a systematic review examining the risk of bipolar disorder recurrence following discontinuation of mood stabilisers, the authors concluded that in women with unstable forms of the disease, the high risk of relapse associated with *rapid* withdrawal of mood stabilisers more than balanced the potential risk to the foetus [80]. This suggests that gradual withdrawal through down-titration of dose is preferable to abrupt discontinuation.

It is possible to conceptualise hypothetical scenarios in which continuation of sodium valproate during pregnancy could be clinically justifiable. For example, if a patient on valproate presents in her final trimester with a history of severe and unstable bipolar disorder, has relapsed during a previous pregnancy and caused significant harm to herself and/or her baby, the risks of discontinuation may outweigh the risks of continuation, though even in such an extreme case, careful dose reduction may also be an option.

In rare situations where sodium valproate prescription continues during pregnancy, use should be restricted to monotherapy [81] and at the lowest dose possible [56, 76].

### A role for shared decision-making?

In view of the new regulations requiring a woman of childbearing age to sign a risk acceptance form if she chooses to continue valproate, informed decision-making is more important than ever.

Shared decision-making is a process whereby health professionals and patients work together to make healthcare choices [82]. When deciding which treatments are appropriate, a discussion should involve the patient, with the clinician guiding them through the benefits and risks in order to make an informed decision [83]. Policymakers have called for increased collaboration between patients and clinicians [84], and patients are more likely to adhere to a healthcare decision if they have been involved in the process [85].

Legare et al. have carried out a narrative review of the literature on shared decision-making, and systematically debunked various myths regarding it. These include the assumptions that it takes longer to carry out in consultations, costs more money, or that it is incompatible with following clinical practice guidelines [82]. The authors also show that contrary to many clinicians’ expectations, vulnerable patients who appear passive tend to gain more clinical benefit than those whose decision is made for them.

There is a wide variety of evidence-based tools to support shared decision-making during the clinical encounter [86], and some of these have been trialled successfully in specific diseases. For example, the *talk model*, developed by Elwyn et al. [87], improves treatment concordance and decision quality in epilepsy management, specifically for situations like pregnancy and medication withdrawal [84]. The model involves three kinds of talk: (1) team talk, where the patient is encouraged to consider different management strategies (e.g. valproate vs. levetiracetam for epilepsy); (2) option talk, where more detailed information about the options is provided (typically using an ‘option grid’ – a matrix structured around questions the patient might ask and covering all potential options for a particular set of circumstances), and (3) decision talk, where the clinician assists the patient with decisions by providing facts, figures and risks [87].

The *talk model* maps well to questions around valproate use in which women who are pregnant (or planning on becoming pregnant). The clinician should firstly present the different management options. These would include: (1) continuing the drug at the current dose, (2) titrating down to a lower dose, (3) discontinuing the drug, or (4) changing to a different medication. To support more detailed conversations, a matrix of options could be offered (see example in Table 5).

**Table 5:** List of frequently asked questions and management options to support shared decision-making regarding valproate use for bipolar disorder before or during pregnancy

Frequently asked questions	Continuing the current dose of valproate	Lowering the dose of valproate	Discontinuing valproate	Changing to another medication
What does it involve?	No change to medication or dose	Over a period of weeks to months, decreasing the amount of valproate	Over a period of weeks to months, gradually stopping valproate	Switching to a different medication (e.g. lamotrigine or an antipsychotic)
What are the risks to me?	Usual side effects of valproate	Usual side effects of valproate, potential for relapse	Higher risk of relapse (depends on a variety of factors – discuss with your clinician), increased risk of puerperal psychosis	Risk of relapse if the other medication is not as effective as valproate; risk of new side effects
What are the risks to my baby?	Congenital malformations (see Table 4) long-term developmental disorders (estimated one in 3)	Reduced risk of congenital malformations and developmental disorders (risk depends on the dose, discuss with your clinician)	Indirect risks, e.g. disinhibition from poorly controlled bipolar disorder (discuss with your clinician)	Some medications are much safer for your unborn baby (specifically lamotrigine, some antipsychotics)
What are the benefits?	You are less likely to relapse or suffer from puerperal psychosis	Your unborn baby will have a lower risk of malformations than if you continue the full dose	Your unborn baby will have the same risk of malformations as the general population	If you can tolerate the new drug, you are less likely to relapse or suffer from puerperal psychosis; the other medication could have adverse effects
Who would benefit most from this?	People with unstable bipolar disorder and frequent relapses who are not controlled on other medication or lower doses of valproate	People with bipolar disorder who are not controlled on other medication	People who have been stable off valproate and do not wish to take other medications during pregnancy	People who are stable on alternatives to valproate

The third step in the talk model would be for the patient to discuss their preferred option with the clinician in detail, which would involve getting a more accurate picture of the risks involved. For example, if the woman chooses to discontinue valproate (column 4 in Table 3) the clinician should outline that this still presents risks to the baby, with the potential for the mother to become mentally unwell and hence perhaps neglect herself and her baby, leading in extreme cases to separation via social services. As noted above, the precise risks of this eventuality will depend on the medical and sociodemographic details of the individual case. It is hoped that in most cases clinician and patient will be able to collaboratively weigh up the pros and cons and achieve a decision that takes account of both mother and baby’s health.

The question of shared decision-making for a teratogenic drug in pregnancy raises an ethical conundrum. Whilst the mother may choose to continue valproate (e.g. if her bipolar disorder or epilepsy is well-controlled on it), the foetus has no chance to express a view. Arguably, a ‘paternalistic’ decision by the clinician to discontinue (or not commence) valproate in a pregnant woman is actually a form of advocacy for a second patient (the foetus). Others might argue that this line of reasoning presupposes that a) the foetus will necessarily come to less harm overall if valproate is not given (in reality the balance of benefits versus harms may be more complex); and b) the mother will be motivated by self-interest rather than taking her unborn child’s needs into account.

### The future

Given the limited evidence base on the precise benefits and harms associated with alternatives to sodium valproate during pregnancy, randomised controlled trials to assess their relative efficacies and safety profiles are justified. Various such trials are ongoing.

Personalised (that is, stratified) medicine offers some potential avenues for further research. One study has demonstrated that specific maternal and foetal genotypes in mice confer a greater susceptibility to the teratogenic effects of valproate [88], and may mean that in the future, some human genotypes could be preselected for continuing (or not continuing) the drug during pregnancy. More speculatively, environmental factors may predict susceptibility to valproate-induced teratogenicity. Ogawa et al. showed that geographical stressors, in particular the location of conception, significantly affected pregnant rats’ sensitivity to teratogenicity during valproate use [89]. However, in general the hope for stratified solutions has greatly exceeded the clinical benefit of such solutions, so we should remain cautious about such options [90].

## Conclusion

The management of bipolar disorder and epilepsy during pregnancy continues to carry complex challenges. Sodium valproate use is a growing issue in women of childbearing age, and patients and clinicians may well be faced with a situation in which they must weigh up the benefits and harms of continuation.

Although the teratogenic and long-term neurodevelopmental effects of valproate are now well established, the current evidence suggests that the recent strengthening of regulatory restrictions on its use are justified. However, it would be premature to ban the use of this drug in women of childbearing age in the UK (since in rare cases the benefits of its use may outweigh potential harms). In the light of the new regulatory framework, we believe that informed, democratic conversations are needed and we have provided an evidence-based framework to support shared decision-making. More research should be undertaken on how shared decision-making plays out in this group of patients, as well as on the efficacy and safety of the various alternatives to sodium valproate in bipolar disorder and epilepsy.

## Abbreviations

ANSM: French National Agency for the Safety of Medicines and Health Products
EURAP: International Registry of Anti-Epileptic Drugs and Pregnancy
MHRA: Medicines and Healthcare Devices Regulatory Agency
NNT: Number Needed to Treat
